# Sld3-MCM Interaction Facilitated by Dbf4-Dependent Kinase Defines an Essential Step in Eukaryotic DNA Replication Initiation

**DOI:** 10.3389/fmicb.2016.00885

**Published:** 2016-06-10

**Authors:** Dingqiang Fang, Qinhong Cao, Huiqiang Lou

**Affiliations:** State Key Laboratory of Agro-Biotechnology, College of Biological Sciences, China Agricultural UniversityBeijing, China

**Keywords:** budding yeast, DNA replication, Sld3/Treslin, DNA helicase, Mcm2-7

## Abstract

Sld3/Treslin is an evolutionarily conserved protein essential for activation of DNA helicase Mcm2-7 and replication initiation in all eukaryotes. Nevertheless, it remains elusive how Sld3 is recruited to origins. Here, we have identified the direct physical association of Sld3 with Mcm2 and Mcm6 subunits *in vitro*, which is significantly enhanced by DDK *in vivo*. The Sld3-binding domain (SBD) is mapped to the N-termini of Mcm2 and Mcm6, both of them are essential for cell viability and enriched with the DDK phosphorylation sites. Glutamic acid substitution of four conserved positively charged residues of Sld3 (*sld3-4E*), near the Cdc45-binding region, interrupts its interaction with Mcm2/6 and causes cell death. By using a temperature-inducible degron (td), we show that deletion of Mcm6 SBD (*mcm6ΔN122*) abolishes not only Sld3 enrichment at early origins in G1 phase, but also subsequent recruitment of GINS and RPA during S phase. These findings elucidate the *in vivo* molecular details of the DDK-dependent Sld3-MCM association, which plays a crucial role in MCM helicase activation and origin unwinding.

## Introduction

The initiation of eukaryotic chromosome replication is spatial-temporally controlled by the assembly and activation of DNA helicase mini-chromosome maintenance (MCM) composed by six paralogous subunits (Mcm2-7) in a multi-step fashion (Remus and Diﬄey, 2009; Masai et al., 2010; Vijayraghavan and Schwacha, 2012; Bell and Kaguni, 2013; Li and Araki, 2013; Tognetti et al., 2014).

From late M to G1 phase, two Mcm2-7 heterohexameric rings are loaded onto each origin as a double hexamer, which results in the assembly of the pre-replication complex (pre-RC) in yeast (Evrin et al., 2009; Remus et al., 2009; Duzdevich et al., 2015; Ticau et al., 2015). Mcm2-7 helicase remains inactive during this licensing step. When cells enter the S phase, the Mcm2-7 complex must undergo multiple sophisticated changes prior to executing the origin unwinding task. First, Sld3, Sld7 and Cdc45 are recruited to the pre-RC and assembled into the Cdc45-MCM-Sld3 (CMS) complex with the elevated levels of Dbf4-dependent kinase (DDK; Labib, 2010; Heller et al., 2011; Tanaka et al., 2011). Second, the S-phase cyclin-dependent kinases (S-CDKs) phosphorylate Sld3 and Sld2 to stimulate their interactions with Dbp11 (the scaffold of the pre-loading complex containing Sld2, GINS and DNA polymerase 𝜀), which leads to the assembly of the pre-initiative complex (pre-IC; Tanaka et al., 2007; Zegerman and Diﬄey, 2007; Muramatsu et al., 2010). Then, Sld3 is replaced by GINS (Go, Ichi, Nii and San, for Sld5, Psf1, Psf2, Psf3), eventually leading to the formation of the active holo-helicase Cdc45-MCM-GINS (CMG) complex (Kanemaki and Labib, 2006; Bruck and Kaplan, 2011; Tanaka et al., 2011; Ilves et al., 2012; Costa et al., 2014; Bruck and Kaplan, 2015b; Sheu et al., 2016).

Firstly discovered in budding yeast as a synthetic lethal mutant with *dpb11-1*, Sld3/Treslin is a conserved initiation factor from fungi to higher eukaryotes (Kamimura et al., 2001; Kumagai et al., 2010; Boos et al., 2011). The carboxy-terminus of Sld3 is phosphorylated by S-CDK and then binds to the amino-terminal BRCT repeats of Dpb11(Tanaka et al., 2007; Zegerman and Diﬄey, 2007), while its amino-terminus comprises a Cdc45-binding domain (CBD) as shown in the crystal structure of Sld3-Cdc45 complex from budding yeast (Itou et al., 2014). During the process of MCM helicase activation, the binding of Sld3-Cdc45 to pre-RC is a pivotal step (Kamimura et al., 2001; Kanemaki and Labib, 2006). The Mcm2-7 complex is thought to be the docking platform for Sld3-Cdc45 recruitment. Mcm2-7 phosphorylation by DDK is also presumed to be involved in Sld3-Cdc45 loading albeit with unknown mechanisms (Labib, 2010; Tanaka et al., 2011; Heller et al., 2011; Bruck and Kaplan, 2015a,b; Yeeles et al., 2015).

In this study, we report that Sld3 is recruited through binding directly to the N-termini of Mcm2 and Mcm6 subunits *in vitro*, which are enriched with the phosphorylation sites of DDK. Indeed, their interactions are pronouncedly enhanced by DDK *in vivo*. According to the crystal structure of Sld3, we are able to identify that four highly conserved positively charged residues of Sld3, near the Cdc45-binding region, are important for associating with Mcm2/6 and cell viability. By utilizing a temperature-inducible degron (td) strategy, we show that the interaction defective mutations in Mcm6 (*mcm6Δ122*) interrupt the origin association of Sld3, but not Mcm2-7 *per se*. The subsequent recruitment of GINS to Mcm2-7 becomes abolished, which in turn compromises the Mcm2-7 helicase-driven origin unwinding as evidenced by the RPA-ChIP analysis. These data provide the detailed mechanism by which Sld3 is recruited to the Mcm2-7 complex in a DDK-dependent manner, which defines an upstream step in helicase activation and replication initiation in eukaryotes.

## Results And Discussion

### Sld3 Interacts with Mcm2-7 in a DDK-Dependent Manner

**FIGURE 1:**
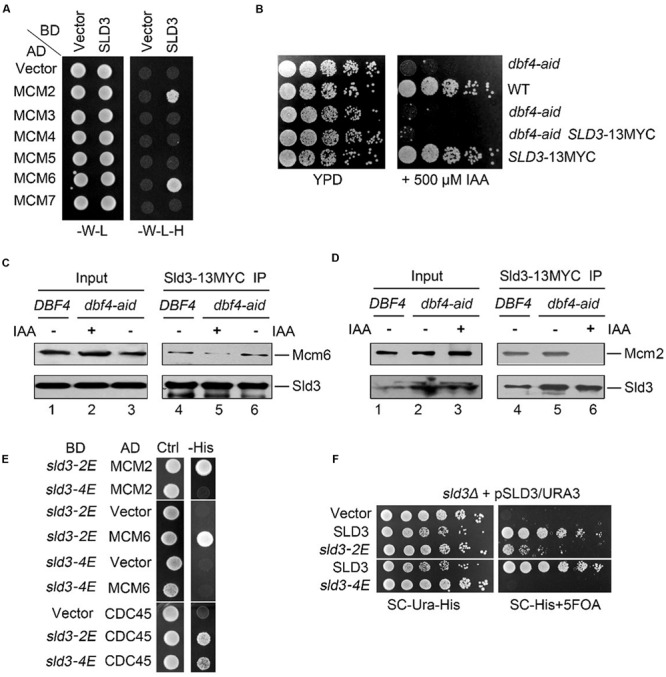
**DDK-dependent Sld3–MCM interaction is essential for viability. (A)** Sld3 displays robust interactions with Mcm2 and Mcm6 subunits in the yeast two hybrid assay. Yeast cells transformed with the indicated plasmids were grown at 30°C on either SC-W-L or SC-W-L-H plates as described in Experimental Procedures. (BD: Gal4 DNA binding domain fusion; AD: Gal4-activation domain fusion). **(B)** An auxin-inducible degron (aid) system to deplete the essential protein Dbf4 in yeast cells. The aid system was turned on by addition of 500 μM IAA. **(C,D)** The interaction between Sld3 and Mcm6 **(C)** or Mcm2 **(D)** is significantly impaired in the absence of Dbf4. Cells were cultured with or without IAA treatment for 2 h. Cell lysates were subjected to Sld3-13MYC immunoprecipitation. The precipitates were analyzed by western blot with the indicated antibodies. **(E)** Glu-substitution of a conserved basic patch at the N-terminus of Sld3 disrupts the Sld3–MCM interaction. **(F)** The MCM-interaction defective mutant *sld3-4E* fails to grow. Plasmid shuﬄing was carried out as described in Experimental Procedures. The strains expressing the indicated plasmid were plated on selective medium, or supplemented with 5-FOA, then grown for 2 days at 30°C.

To investigate the molecular mechanism of Sld3 recruitment and CMS assembly, we set out to characterize the protein–protein interactions mediated by Sld3. In the yeast two hybrid assay, Sld3 interacted with Mcm2 as previously reported (**Figure 1A**; Herrera et al., 2015). Meanwhile, we found that Sld3 also displayed robust interaction with Mcm6 subunit.

Since DDK has been shown to be involved in Sld3 recruitment (Heller et al., 2011; Tanaka et al., 2011), we next tested whether DDK promotes Sld3–MCM interaction. To this end, we adopted an auxin-inducible degron (aid) method to deplete cellular Dbf4 proteins (Nishimura et al., 2009). When 500 μM indole-3-acetic acid (IAA) was supplemented in the medium, yeast cells carrying the carboxy-terminal aid tagged *DBF4* (*dbf4-aid*) failed to grow, suggesting the efficient degradation of Dbf4 proteins which are essential for cell growth (**Figure 1B**). Meanwhile, *SLD3* gene was tagged with 13MYC at the genomic locus, which did not alter the normal cell growth. Sld3-13MYC was immunoprecipitated with an anti-MYC antibody and detected by western blotting following SDS-PAGE. In the absence of IAA, Mcm6 was detected in the immunoprecipitate of Sld3-13MYC from *dbf4-aid* cells as well as from wild-type (WT) cells (**Figure 1C**, lanes 4 and 6). These results corroborate the physical association between Sld3 and Mcm6 *in vivo*. However, after IAA treatment for 2 h, the levels of Mcm6 co-precipitated with Sld3 were significantly decreased (**Figure 1C**, compare lane 5 to 6). In an independent experiment, co-precipitation of Mcm2 with Sld3 was also abrogated by the auxin-induced Dbf4 depletion (**Figure 1D**, compare lane 6 to 5). These data indicate that Sld3-MCM association is facilitated by DDK, through catalyzing the phosphorylation of Mcm2-7 subunits as demonstrated in a very recent study (Deegan et al., 2016).

### Sld3-MCM Interaction is Essential for Cell Growth

Sld3 does not bear any apparent known phosphopeptide-binding motifs. Interestingly, it comprises two conserved positively charged regions close to each other as revealed by the crystal structure (Itou et al., 2014). One basic patch (Sld3-BP1, a.a. 301–330) mediates the interaction with an acidic region of Cdc45. Interestingly, the second basic patch (Sld3-BP2, K181, R186, K188, R192, K404, K405) is not involved in Cdc45 binding, but is also important for cell growth albeit with unknown function (Itou et al., 2014). We hypothesized that Sld3-BP2 might be involved in binding to phosphorylated MCM subunits. To test this, we first constructed the Glu-substitution mutants of the positively charged residues within Sld3-BP2. In the yeast two hybrid assay, the interaction with either Cdc45 or Mcm2/6 was not dramatically affected in the Sld3-2E(K188E, R192E) mutants (**Figure 1E**). Sld3-4E (K188E, R192E, K404E, K405E) also retained positive interaction with Cdc45 (**Figure 1E**, lower panel), which is consistent with the previous co-purification results (Itou et al., 2014). In contrast, Sld3-4E completely lost the interactions with both Mcm2 and Mcm6 (**Figure 1E**). These results indicate that the DDK-dependent Sld3–MCM interaction is mainly mediated by the positively charged Sld3-BP2 close to the Cdc45 binding interface, which might be an evolutionarily conserved event given that these basic residues are highly conserved between Sld3 and Treslin.

We next examined the physiological role of Sld3–MCM interaction in yeast. Since *SLD3* is essential for cell growth, we constructed the *sld3* mutants via plasmid shuﬄing. Briefly, WT *SLD3* was introduced by a plasmid with *URA3* selective marker. The genomic copy of *SLD3* was then knocked out. The *sld3* mutants were expressed from a *HIS3* plasmid. The *URA3* plasmid can be counter-selected on a 5-fluoro-orotic acid (5-FOA) plate. Therefore, cell growth on the 5-FOA plates reflects the physiological function of the remaining *sld3* copy in the *HIS3* plasmid. Strikingly, *sld3-4E* was not able to support cell growth, whereas *sld3-2E* showed moderate sick growth (**Figure 1F**), correlating with their ability to interact with MCM. These data suggest that Sld3–MCM interaction is essential for cell viability.

### Sld3 Binds Directly with the N-Termini of Mcm2 and Mcm6

**FIGURE 2:**
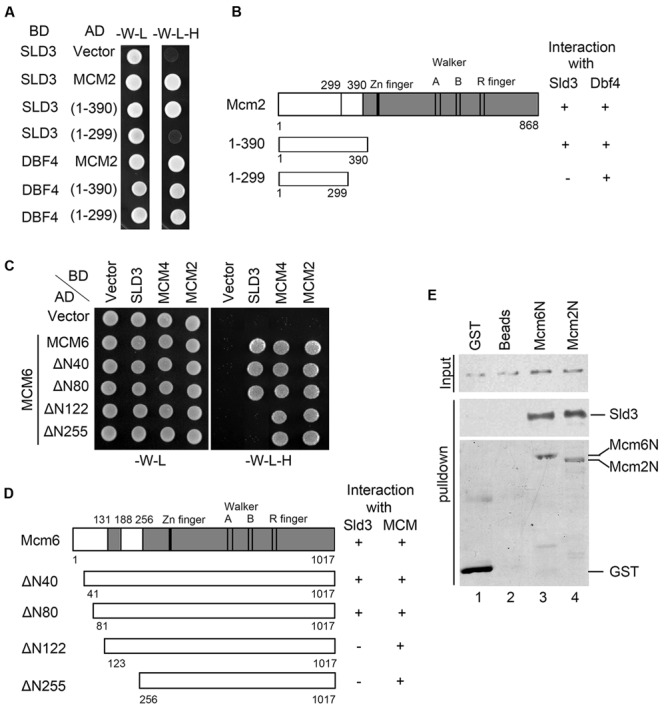
**Sld3 binds directly to the N-terminal regions of Mcm2 and Mcm6. (A,B)** Sld3 interacts with a short region near the Mcm2 N-terminus (300–390) in the yeast two hybrid assay as described in **Figure 1A**. **(C)** Mapping the domain of Mcm6 required for interaction with Sld3. Mcm6 truncations were constructed and tested in the yeast two hybrid assay as described above. **(D)** A summary of Mcm6 truncations and their ability to interact with Sld3 and other MCM subunits, respectively. **(E)** The N-termini of Mcm6 and Mcm2 are sufficient to bind directly to Sld3 *in vitro*. Purified recombinant GST-Mcm6 (1–439) or GST-Mcm2 (1–390) and 6His-Sld3 were incubated with glutathione Sepharose beads in the binding buffer containing 1 μg/μl BSA. The protein bands were detected by immunoblots with anti-His and anti-GST antibodies, respectively.

Then, we mapped the Sld3-binding domain (SBD) in Mcm2. Through construction of a series of Mcm2 truncations, we identified that a small region (a.a. 300–390) close to its N-terminus was required for interaction with Sld3 (**Figure 2A**). Moreover, the very N-terminal 299 amino acids were sufficient to bind Dbf4, which is consistent with a previous report that devoid of the N-terminal 63 amino acids in Mcm2 abolishes the interaction with Dbf4 (Ramer et al., 2013). These results indicate that Sld3 and Dbf4 interact with two adjacent regions within the Mcm2 amino terminus (**Figure 2B**). Similarly, when the N-terminal 122 amino acids were deleted, Mcm6 lost the interaction with Sld3. Meanwhile, the interactions of Mcm6 with its neighbor MCM subunits (Mcm2 or Mcm4) were not affected (**Figures 2C,D**), indicating that the SBD of Mcm6 is separable from the interface of the Mcm2-7 hexameric complex. When pull-down experiments were conducted with purified recombinant proteins, Sld3 was successfully detected together with both GST-Mcm2N (1–390) and GST-Mcm6N (1–439), indicating a direct physical association between Sld3 and Mcm2/6 N-termini (**Figure 2E**). Taken together, these data suggest that both N-termini of Mcm2 and Mcm6 mediate interaction with Sld3, which is enriched with the DDK phosphorylation sites (Randell et al., 2010; Sheu and Stillman, 2010). These results are in agreement with the notion that Sld3–MCM interaction can be facilitated by DDK as shown in **Figure 1** and other studies (Heller et al., 2011; Tanaka et al., 2011; Deegan et al., 2016).

### Mcm6 SBD is Indispensable for Replication Initiation

**FIGURE 3:**
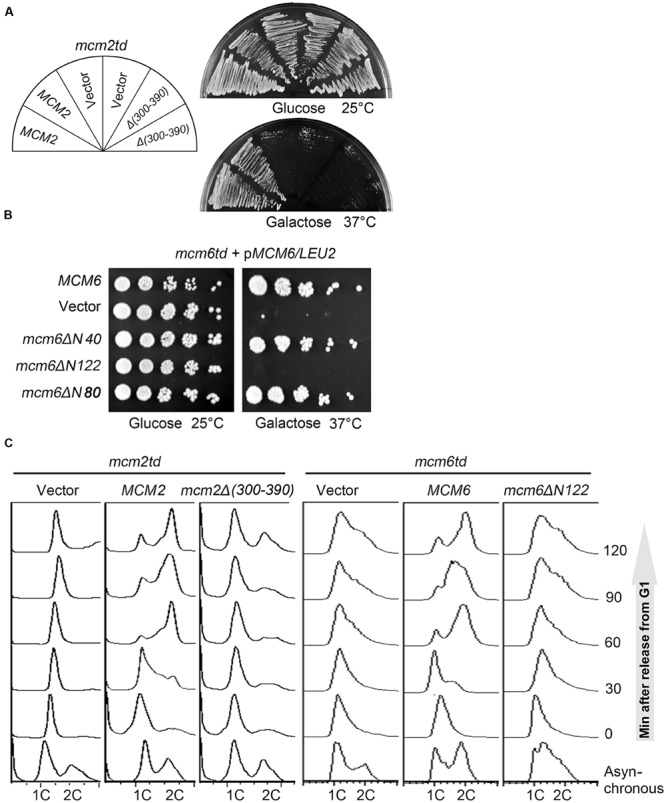
**The SBDs of Mcm2 and Mcm6 are indispensable for replication initiation and cell viability. (A)** Yeast cells are not able to grow in the absence of the Sld3 interaction motif of Mcm2. A plasmid expressing the indicated *MCM2* alleles was introduced into the *td-MCM2* background strain. The endogenous Mcm2 was depleted by a temperature-inducible degron at 37°C in the presence of galactose. **(B)** The N-terminal 122 amino acids deletion of Mcm6 causes cell death. 5-fold serial dilutions of *td-MCM6* cells harboring the indicated plasmids were spotted on either glucose or galactose plates. The endogenous Mcm6 was depleted as described above. **(C)** Disruption of the Sld3 interaction regions of Mcm2 or Mcm6 compromises the S phase entry. Cells were released from α factor synchronization and analyzed for DNA content by flow cytometry.

Next, we asked whether the Sld3 interaction defective mutations in Mcm2/6 affect normal cell growth. To this end, we adopted a temperature-inducible degron (td) to deplete the endogenous Mcm2 proteins (Kanemaki et al., 2003). The expression of ubiquitin ligase E3 Ubr1 for td-labeled protein degradation is under control of a galactose-inducible promoter. The separation-of-function mutations were introduced in a plasmid copy of *MCM2*. When cells were switched to the galactose plates incubated at 37°C, the endogenous td-Mcm2 proteins were degraded and resulted in cell death (**Figure 3A**). Strikingly, the lethality could be rescued by an extra copy of WT *MCM2*, but not an *mcm2Δ(300–390)* allele. Similarly, a Mcm6 mutant devoid of SBD, the N-terminal 122 amino acids (*mcm6ΔN122*), was not able to support yeast growth either (**Figure 3B**). These results are consistent with the phenotype of the interaction defective *Sld3-4E* mutants described in **Figure 1F**. Recently, Itou et al reported a hetero-tetrameric structure of Sld3-Sld7 (Itou et al., 2015), which provides one possible scenario that two Sld3 molecules bind to Mcm2 and Mcm6, respectively. Putting together, these data suggest that both Mcm2 and Mcm6 N-termini mediated interactions with Sld3 are essential requirements for cell viability.

Since both MCM and Sld3 play essential roles in DNA replication initiation. We then examined whether the lethality of the Sld3–MCM interaction defective mutant is due to the failure in DNA replication by flow cytometry. Notably, both *mcm2Δ(300–390)* and *mcm6ΔN122* mutant cells were significantly compromised in S phase progression, thus implying that both Mcm2- and Mcm6- mediated interactions with Sld3 are crucial for replication initiation (**Figure 3C**).

### Mcm6 SBD Participates in Sld3 Recruitment and Origin Unwinding

**FIGURE 4:**
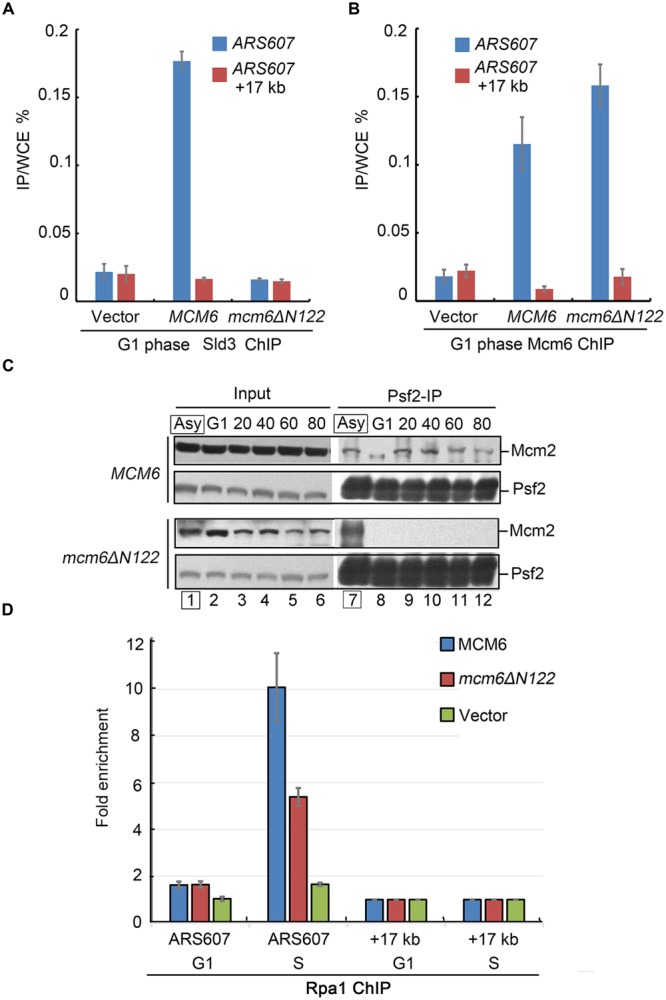
**Sld3 recruitment and GINS–MCM interaction are compromised in *mcm6ΔN122.* (A)** Sld3 recruitment at early origin *ARS607* is abrogated in *mcm6ΔN122*. Sld3-13MYC ChIP-qPCR was conducted as described in Experimental Procedures. A non-origin region, 17 kb downstream of *ARS607*, is detected as a negative control. **(B)** Enrichment of Mcm6 at *ARS607* is not affected in *mcm6ΔN122*. Anti-Mcm6 ChIP was conducted basically as described above. **(C)** Interaction between Psf2 (a GINS subunit) and Mcm2 is abolished in the absence of the Sld3 binding module of Mcm6. After cultured to OD600 0.3 at 23°C, yeast cells were arrested by α-factor in G1 at 23°C for 3 h and released into galactose media with α-factor at 37°C for 1 h, then released into fresh galactose media for the indicated times (S phase). The endogenous td-Mcm6 proteins were depleted when cells were shifted to 37°C during synchronization, but not in the asynchronized samples (Asy in the box). Cell lysates were subjected to Psf2 immunoprecipitation and analyzed by western blot. **(D)** The origin association of Rfa1 becomes compromised in the interaction defective mutant *mcm6ΔN122*. Cells were arrested in G1 and the endogenous td-Mcm6 proteins were depleted as above. After released into fresh galactose media for another 20 min (early S phase), cells expressing WT MCM6 or *mcm6ΔN122* were withdrawn from the culture and subjected to Rfa1-13MYC ChIP-qPCR as described in **(A)**.

To investigate the exact role of Mcm6 N-terminus medicated interaction with Sld3, we carried out three sets of experiments. First, a chromatin immunoprecipitation (ChIP) assay was performed to evaluate the enrichment levels of Sld3 at early origins in G1-arrested cells. The endogenous Sld3 proteins carrying a 13MYC tag were immunoprecipitated by an anti-MYC antibody from the formaldehyde cross-linked cell lysates. The co-immunoprecipitated DNA was analyzed by quantitative PCR (ChIP-qPCR). A significant amount of *ARS607* DNA was detected in WT cells, but not in *mcm6ΔN122* mutant (**Figure 4A**). Meanwhile, the origin localization of Mcm6 itself was not changed after deletion of Mcm6 SBD (**Figure 4B**). These results indicate that the origin recruitment of Sld3 is inhibited when Mcm6 loses Sld3 binding capacity.

The recruitment of Sld3 to pre-RC has been shown to be required for subsequent GINS recruitment (Kanemaki and Labib, 2006; Bruck and Kaplan, 2015a,b). Therefore, Psf2, a subunit of GINS complex, was examined for its association with MCM during the cell cycle. G1-arrested cells were harvested every 20 min after release into the S phase. Cell lysates were subjected to anti-Psf2 immunoprecipitation. In WT, the amounts of Mcm2 co-precipitated with Psf2 were gradually increased with the S phase progression (**Figure 4C**). Strikingly, there was barely detectable Mcm2 in the precipitate of *mcm6ΔN122* (**Figure 4C**). On the contrary, if the endogenous Mcm6 was not depleted like in the asynchronized cells, Mcm2 was detected normally (lane 7). These results suggest a severe defect in the assembly of active CMG.

Third, we asked whether origin unwinding by MCM helicase depends on Mcm6 SBD. When duplex origin DNA is unwound by activated MCM helicase, the single-stranded DNA (ssDNA) is immediately protected by RPA. Therefore, RPA localization at origins can reflect the MCM-created ssDNA levels *in vivo*. The lysates were prepared from cells synchronized at G1 or early S phase (20 min after release from G1). Rfa1-3HA was precipitated from the lysates of G1 or S phase cells by an anti-HA antibody. Coprecipitated DNA was measured by qPCR. The endogenous Mcm6 was depleted by the td degron as described above. In the presence of WT *MCM6*, the relative enrichment of Rpa1 at early origin *ARS607* substantially increased after release from G1 phase for 20 min (**Figure 4D**). Nevertheless, in *mcm6ΔN122*, the signals of Rpa1 at this origin were about 50% lower compared to the ones in WT, indicating a severe defect in the origin recruitment of RPA (**Figure 4D**). These results suggest that origin unwinding by MCM helicase activity is dependent on the Sld3–MCM interaction.

In this study, we provide the molecular details of the DDK-dependent recruitment of Sld3 to the MCM complex. These findings, together with the previous notion of Sld3-dependent Cdc45 and GINS loading (Kanemaki and Labib, 2006; Bruck and Kaplan, 2015b; Herrera et al., 2015; Deegan et al., 2016), shed new light on the assembly of the CMS complex, which defines a crucial transient step upstream of the recruitment of GINS and assembly of the active form of the replicative helicase, the CMG complex, for duplex DNA unwinding and replication initiation.

## Experimental Procedures

### Yeast Strains and Basic Methods

The yeast strains used in this study are derived from W303-1a (**Table 1**) and constructed basically as previously described (Lou et al., 2008). Plasmids constructed for this study are listed in **Table 2** and validated by sequencing. Yeast two-hybrid, plasmid shuﬄing, cell synchronization and flow cytometry analysis were performed as described in (Quan et al., 2015).

**Table 1:** Yeast strains in this study.

Strain	Genotype	Source
W303-1a	*MATa trp1-1 ura3-1 his3-11,15 leu2-3,112 ade2-1 can1-100 RAD5*	In stock
YDF101	*SLD3-13MYC (HIS3)*	This study
YKL76	*mcm6-td (URA3) ubr1::GAL-UBR1 (HIS3)*	From Karim Labib
YKL69	*mcm2-td (URA3) ubr1::GAL-UBR1 (HIS3)*	From Karim Labib
9077	*dbf4-aid::natNT2*	From Toyoaki Natsume
YDF102	*dbf4-aid SLD3-13MYC (HIS3)*	This study
YDF103	*dbf4-aid SLD3-5FLAG (LEU2)*	This study
YDF104	*sld3::NAT [pRS316-SLD3]*	This study
YDF105	*sld3::NatMX [pRS316-SLD3] [pRS313-SLD3]*	This study
YDF106	*mcm6-td [pMCM6/LEU2]*	This study
YDF110	*sld3::NatMX [pRS316-SLD3][pRS313-sld3-2E]*	This study
YDF111	*sld3::NatMX [pRS316-SLD3] [pRS313-sld3-4E]*	This study
YDF112	*mcm6-td [pmcm6ΔN40/LEU2]*	This study
YDF113	*mcm6-td [pmcm6ΔN80/LEU2]*	This study
YDF107	*mcm6-td [pmcm6ΔN122/LEU2]*	This study
YDF115	*mcm6-td [pmcm6ΔN255/LEU2]*	This study
YDF108	*mcm6-td RFA1-3HA(G418)[pMCM6/LEU2]*	This study
YDF109	*mcm6-td [pmcm6ΔN122/LEU2] RFA1-3HA(G418)*	This study
YDF116	*mcm2-td [pMCM2/LEU2]*	This study
YDF117	*mcm2-td [pmcm2Δ300–390/LEU2]*	This study

**Table 2:** Plasmids used in this study.

Plasmid	Base plasmid/Genotype	Source
pET28a-SLD3	kan^r^ 6His- SLD3	This study
pET28a-mcm2(1–390)	kan^r^ 6His- mcm2(1–390)	This study
pGEX-4T-1-SLD3	amp^r^ GST- SLD3	This study
pGEX-4T -1-sld3(1–548)	amp^r^ GST- sld3(1–548)	This study
pGEX-4T -1-mcm2(1–390)	amp^r^ GST- mcm2(1–390)	This study
pGEX-4T -1- mcm6(1–496)	amp^r^ GST- mcm6(1–496)	This study
pGADT7-MCM2	amp^r^/LEU2 GAL4-AD-MCM2	This study
pGADT7-MCM3	amp^r^/LEU2 GAL4-AD-MCM3	This study
pGADT7-MCM4	amp^r^/LEU2 GAL4-AD-MCM4	This study
pGADT7-MCM5	amp^r^/LEU2 GAL4-AD-MCM5	This study
pGADT7-MCM6	amp^r^/LEU2 GAL4-AD-MCM6	This study
pGADT7-MCM7	amp^r^/LEU2 GAL4-AD-MCM7	This study
pGBKT7-MCM4	kan^r^/TRP1 GAL4-BD-MCM4	This study
pGBKT7-MCM2	kan^r^/TRP1 GAL4-BD-MCM2	This study
pGADT7- mcm6ΔN40	amp^r^/LEU2 GAL4-AD-mcm6ΔN40	This study
pGADT7- mcm6ΔN80	amp^r^/LEU2 GAL4-AD-mcm6ΔN80	This study
pGADT7- mcm6ΔN122	amp^r^/LEU2 GAL4-AD-mcm6ΔN122	This study
pGADT7- mcm6ΔN255	amp^r^/LEU2 GAL4-AD-mcm6ΔN255	This study
pGBKT7-SLD3	kan^r^/TRP1 GAL4-BD-SLD3	This study
pGBKT7-sld3-2E(K188E,R192E)	kan^r^/TRP1 GAL4-BD-sld3-2E	This study
pGBKT7-sld3-4E(K188E,R192E, K404E,K405E)	kan^r^/TRP1 GAL4-BD-sld3-4E	This study
pGADT7-CDC45	amp^r^/LEU2 GAL4-AD-CDC45	This study
pRS313-SLD3	amp^r^/HIS3 SLD3	This study
pRS313-sld3-2E	amp^r^/HIS3 sld3-2E	This study
pRS313-sld3-4E	amp^r^/HIS3 sld3-4E	This study
pRS316-SLD3	amp^r^/URA3 SLD3	This study
pGBKT7-DBF4	kan^r^/TRP1 GAL4-BD-DBF4	This study
pGADT7-mcm2(1–299)	amp^r^/LEU2 GAL4-AD-mcm2(1-299)	This study
pGADT7-mcm2(1–390)	amp^r^/LEU2 GAL4-AD-mcm2(1–390)	This study
pRS315-MCM2	amp^r^/LEU2 MCM2	This study
pRS315-mcm2Δ300–390	amp^r^/LEU2 mcm2Δ300–390	This study
pRS315-MCM6	amp^r^/LEU2 MCM6	This study
pRS315-mcm6ΔN40	amp^r^/LEU2 mcm6ΔN40	This study
pRS315-mcm6ΔN80	amp^r^/LEU2 mcm6ΔN80	This study
pRS315-mcm6ΔN122	amp^r^/LEU2 mcm6ΔN122	This study
pRS315-mcm6ΔN255	amp^r^/LEU2 mcm6ΔN255	This study

### Immunoprecipitation (IP)

Immunoprecipitation (IP) was carried out basically as described previously with some modifications (Lou et al., 2008). Briefly, yeast cultures were arrest at G1 phase with α-factor for 3 h. Cells (4 × 10^8^) were crosslinked and collected and lysed at 4°C with glass beads (BeadBeater) in IP buffer [45 mM HEPES, pH 7.2, 150 mM NaCl, 1 mM EDTA, 10% glycerol, 0.2% NP-40, 2 mM DTT, 1 mM PMSF, 1 × Protease Inhibitor Cocktail tablet (Roche), 1 × PhosSTOP tablet (Roche)]. Whole cell extract was mixed with 2 μl indicated antibodies and rotated for 3 h at 4°C, and then 20 μl of protein G beads were added and incubated for another hour. Beads were then washed three times with 1 ml IP buffer and boiled in 50 μl SDS-sample buffer. Western analysis was performed to detect specific proteins. Blots were probed with the indicated antibodies in phosphate-buffered saline containing 0.1% Tween and 2% dried milk. 9E10 (1:1000) was used to detect an MYC tag, M2 (1:1000) to detect Flag-tagged proteins, 12CA5 (1:1000) to detect an HA epitope. Polyclonal sera against Mcm2 (1:10000) and Mcm6 (1:10000) was used to detect corresponding proteins.

### Protein Expression, Purification

All recombinant proteins were overexpressed in *Escherichia coli* BL21 (DE3) CodonPlus RIL (Stratagene). Cells were sonicated in lysis buffer containing 1% Triton X-100. 6His-Sld3 was purified by Ni^2+^ columns (GE Healthcare). The elution was concentrated to 3 mg/ml in the storage buffer 15 mM Tris-HCl (pH 8.0), 150 mM NaCl, 1 mM dithiothreitol, and 15% glycerol. GST-Mcm2, GST-Mcm6 and their derivatives were expressed and purified as previously described (Quan et al., 2015).

### GST-Pull Down

For GST pull-downs, 2 μg 6His-Sld3 and 5 μg GST-Mcm6 (or GST-Mcm2) were incubated with glutathione-Sepharose beads in the presence of binding buffer (40 mM Tris-HCl, pH7.5, 100 mM NaCl, 0.1 mM EDTA, 10% glycerol, 0.1% Triton X-100, 1 mM DTT, 1 mg/ml BSA, 1 mM PMSF, and protease inhibitors) for 1 h at 4°C. The glutathione agarose beads were washed extensively and bound proteins were separated on 8% SDS-PAGE gels. Blots were probed with monoclonal antibody against GST (1:1000) or 6His (1:1000).

### Chromatin Immunoprecipitation (ChIP-qPCR)

ChIP experiments were performed with extracts of formaldehyde cross-linked cells, using 1.0 μg of purified anti-Sld3 or anti-Mcm6 antibodies (Tanaka et al., 2011). After The DNA fragments in precipitates were quantified by real-time PCR for each genomic locus. The ratio of immunoprecipitated DNA to total DNA in input was normalized and shown as fold enrichment (Natsume et al., 2013).

## Author Contributions

HL and DF conceived and designed research. DF and QC performed experiments. HL analyzed the data and wrote the paper with inputs from all other authors.

## Conflict of Interest Statement

The authors declare that the research was conducted in the absence of any commercial or financial relationships that could be construed as a potential conflict of interest.
